# Hand-Foot Syndrome Presentation Post-Capecitabine Treatment in a Black Patient

**DOI:** 10.7759/cureus.26891

**Published:** 2022-07-15

**Authors:** Allison E Whorton, Abrahim N Razzak, Pinky Jha

**Affiliations:** 1 School of Medicine, Medical College of Wisconsin, Milwaukee, USA; 2 Internal Medicine, Medical College of Wisconsin, Milwaukee, USA

**Keywords:** emollient, diagnosis criteria, metaplastic breast cancer, cutaneous hyperpigmentation, chemotherapy response, chemotherapy-related toxicity, capecitabine side effects

## Abstract

Palmar-plantar erythrodysesthesia, commonly known as hand-foot syndrome (HFS), is a side-effect of cancer chemotherapeutic agents such as capecitabine. Patients with HFS oftentimes present with palmoplantar numbness, tingling, burning pain, and/or hyperpigmentation; in advanced grades, blistering and ulceration may occur. In this article, we present the case of a Black patient with grade 1 HFS post-capecitabine treatment for metastasized breast cancer. Prompt recognition for atypical HFS symptom presentation in people of color and discontinuation of capecitabine with supportive treatment can prevent progression to grade 2+ HFS that limits activities of daily living (ADLs).

## Introduction

This case was previously presented as a meeting poster at the 2022 Annual Meeting of the Society of General Internal Medicine (SGIM) on Friday, April 8, 2022.

Palmar-plantar erythrodysesthesia, commonly known as hand-foot syndrome (HFS), is an adverse side effect event that can occur with many chemotherapeutic agents, including high-dose methotrexate, cytarabine, and especially capecitabine [1]. Capecitabine, an oral FDA-approved prodrug of 5-fluorouracil (5-FU) that inhibits DNA synthesis, is effective in treating metastatic colorectal and breast cancers [2]. However, HFS of any grade is reported to affect 43%-71% of patients treated with single-agent capecitabine chemotherapy [3]. Pathogenesis is poorly understood due to differences in mechanisms of action for each drug associated with HFS. Fluoropyrimidine-induced HFS is attributed to the accumulation of 5-FU metabolites broken down by thymidine phosphorylase, found in high concentrations on the palms. At the same time, capecitabine-activated cyclooxygenase-2 (COX-2) inflammatory pathways cause vasodilation, edema, and leukocyte infiltration [4]. HFS initially presents with palmoplantar numbness, tingling, burning pain, or hyperpigmentation on the lateral and distal fat pads of the palms. However, in advanced grades, the pigmentation can spread to the soles of feet, alongside blistering and ulceration [5]. While not lethal, HFS can affect the quality of life and is designated from grade 0 to grade 3 depending on symptom severity. According to the National Cancer Institute, limitation of activities of daily living (ADLs) occurs in grades 2 and 3 [5]. Here we present the case of a Black patient who presented with HFS symptoms after capecitabine treatment for metastatic breast cancer.

## Case presentation

A 39-year-old female with a significant medical history of stage IV breast cancer with metastatic lesions to the brain, lymph nodes, and liver treated with ado-trastuzumab, capecitabine, and tucatinib, bipolar disorder, post-traumatic stress disorder, and herpes simplex virus II infection presented to the ED in November 2021 with chest pain, shortness of breath (SOB), and generalized weakness.

She had extensive management history for metastatic cancer, first diagnosed five years prior to presentation with ER+, PR+, HER2+ left breast multicentric moderately differentiated invasive carcinoma. After undergoing a left breast mastectomy three years before the presentation and extensive chemotherapy thereafter, she was diagnosed with a recurrence of grade 3 invasive ductal carcinoma on her left axilla two years before presentation. Follow-up CT and PET scans showed metastasized chest wall masses, sternal bone lesions, and liver lesions. This patient was also hospitalized multiple times that year for S/P Gamma Knife radiation of a solitary metastasized brain lesion. At the time of presentation, the patient reported decreased appetite and generalized weakness with arthralgias four days prior. She also noted right-sided chest pain that radiated to her right scapula and arm associated with SOB. While she reported associated intermittent headaches, nausea, diarrhea, and generalized abdominal pain, she denied any sick contacts, dysuria, bright red blood per rectum, or increased urinary urgency/frequency.

**Figure 1:**
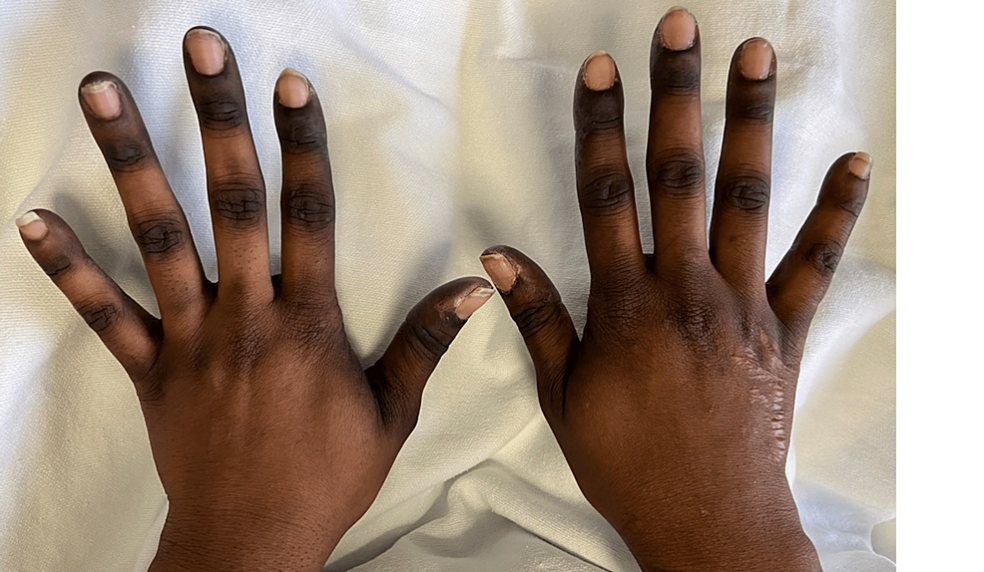
Dark discoloration on creases and joints of the dorsal surface of bilateral hands.

**Figure 2:**
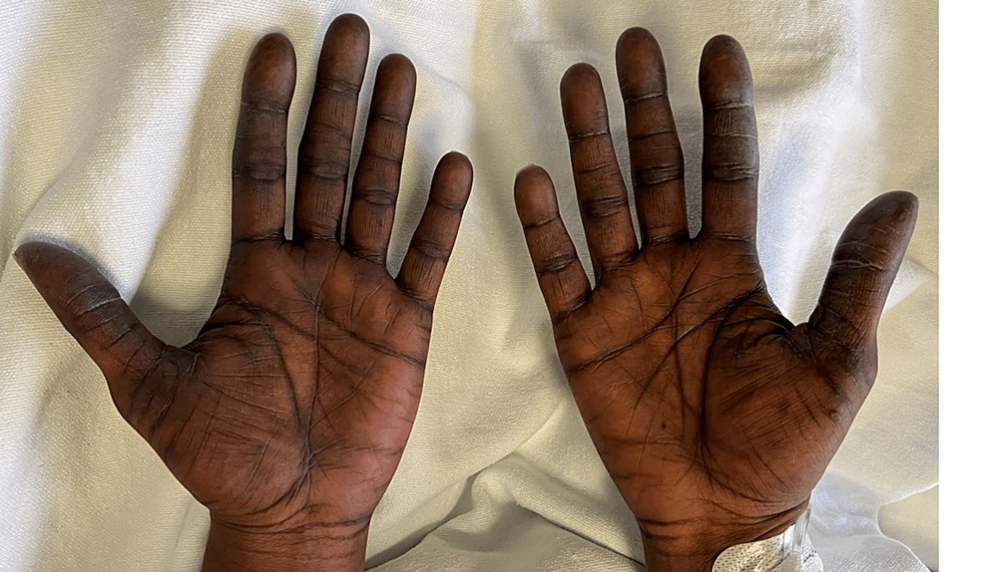
Dark discoloration on creases and joints of the palmar surface of bilateral hands.

**Figure 3:**
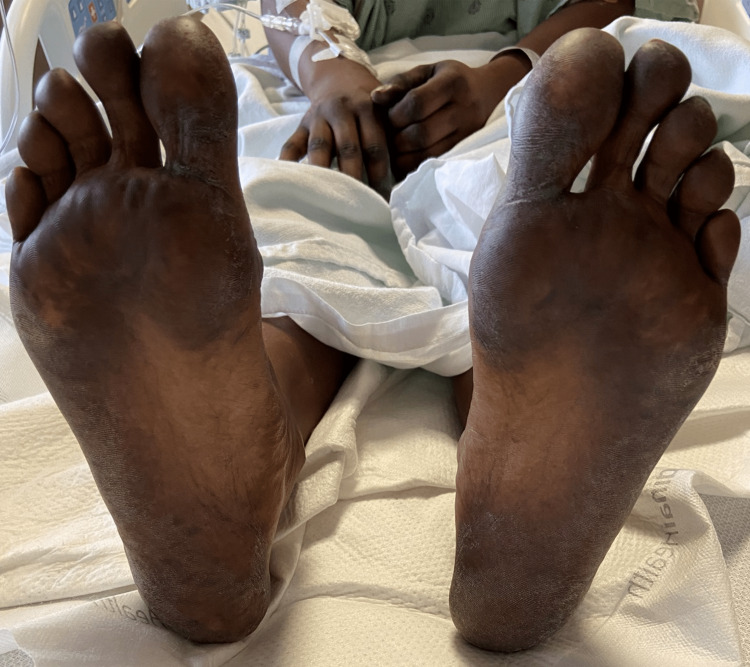
Dark discoloration on the plantar surface of bilateral feet.

Upon work up at the ED and later admission to the hospital, she was found to have an isolated subsegmental pulmonary embolism in the right lower lobe, with no evidence of right heart strain. She was started on heparin and transitioned to therapeutic apixaban when found to be medically stable for discharge without SOB. However, on physical exam, she had incidental findings of dark black discoloration on the creases of palms and soles of feet bilaterally (Figures 1-3). In addition, the patient reported associated paresthesia and swelling.

As a result, all chemotherapeutics were withheld for the duration of her stay of two days while an oncology consult was called due to concern for medication side effects. The oncology team immediately identified the symptoms of HFS, discontinued capecitabine alone, and recommended aggressive cutaneous hydration with thick emollients. In addition, the patient’s chronic peripheral neuropathy may have been partly due to previous docetaxel therapy and may have also impacted the progression of HFS symptoms. She was discharged alongside recommendations to see an outpatient neurooncologist specialist.

## Discussion

This case demonstrated a grade 1 severity HFS reaction likely to capecitabine, with hyperpigmentation symptoms of bilateral upper and lower extremities. However, the exact pathogenesis of HFS is still unknown [5].

Differential diagnoses when HFS is suspected include allergic reactions, contact dermatitis, erythromelalgia, and hand-foot skin reaction (HFSR), amongst others [5]. Given the uncommon physical presentation of HFS, it is often difficult to identify it, leading to continued exposure to the causative medication, which may lead to progression of HFS severity. Alternatively, delays in diagnosis may lead to holding all chemotherapeutics, which can prolong the patient’s metastatic cancer treatment protocol. Effective treatment measures include topical medications (analgesics, corticosteroids, emollients), chemotherapy dose reductions, and switching to other drugs of the same class with lower HFS rates [5]. While this patient’s symptoms were resolved with discontinuation of capecitabine alone, it is not ideal as capecitabine is still a proven effective treatment for both metastatic colorectal cancer and breast cancer [2]. In addition, prophylaxis for HFS is an evolving option within the oncologic specialty; for example, while pyridoxine and topical urea/lactic acid are not associated with decreases in capecitabine-induced HFS, celecoxib has potential prophylactic efficacy [6]. Regardless, the final decision for a treatment protocol should be conducted on a case-by-case basis depending on the patient’s circumstances and presentation. In our case, the patient stopped capecitabine and transitioned to tucatinib at her follow-up oncology visits.

Atypical presentations of HFS for patients with African heritage post-capecitabine treatment have previously been published [7-9]. Our case was unique compared to other cases found in literature as it presented a grade 1 HFS in a Black patient treated for metastatic breast cancer instead of colorectal cancer or GI pathologies. One case presented a grade 2-3 HFS with blistering and ulceration of the extremities [7]. Another report compared three non-Hispanic White HFS cases and Black HFS cases of varying severity grades, concluding that there was a shorter time of progression of severity grades for Black patients from grade 1 to grade 3 HFS [8, 9]. Saif MW and Sandoval A proposed a revision in the staging criteria of HFS symptom manifestation for Black patients [9] (Table 1).

**Table 1:** Proposed Saif MW and Sandoval A criteria for HFS symptom severity in Black patients. Source: [9]

Grade	Manifestation in Black patients
1	Hyperpigmentation of palms and soles
2	Thickening of skin of palms and soles with pain and loss of function
3	Ulceration, dermatitis, or desquamation

In this report, the symptoms aligned with this new proposed grading system in which our patient demonstrated hyperpigmentation of palms and soles (grade 1 HFS) as opposed to the previous grading system looking for presence of dermatitis or erythema without pain [5, 9]. It is also significant to note that one retrospective study conducted by Brazelton A et al. concluded that Black patients had higher success with chemotherapeutic dose reductions as a modem for HFS treatment compared to non-Hispanic Caucasian patients [10]. Therefore, early diagnosis of grade 0 or grade 1 severity HFS may allow prompt and safer treatment modification.

## Conclusions

HFS, while rare in the general populace, is a side effect found in patients undergoing treatment with chemotherapeutic agents, especially capecitabine. While HFS may present atypically in patient populations with darker skin complexions and may be difficult to distinguish from other etiologies, HFS should still be suspected when there is dysesthesia, numbness, and hyperpigmentation along the fat pads of palms and soles of feet. These symptoms can then be treated with topical medications and augmentation of the chemotherapeutic treatment protocol. We hope that this case presentation adds to the growing literature of recommended revisions for HFS symptoms severity criteria in the Black patient population and can act as a diagnostic resource for spotting symptoms earlier in HFS management.
